# Dietary Beliefs and Habits in Pediatric IBD: Insights from a Single-Center Survey

**DOI:** 10.3390/nu18162675

**Published:** 2026-08-16

**Authors:** Dóra Dohos, Emese Kasznár, Anna Karoliny, Dorina Bajzát, Ágnes Eszter Tímár, Judit Szentannay, András Szabó, Eszter Gombos, Katalin Eszter Müller

**Affiliations:** 1 Heim Pál National Pediatric Institute, Üllői Street 86, 1089 Budapest, Hungary; dohos.dora@gmail.com (D.D.);; 2 Centre for Translational Medicine, Semmelweis University, Baross Street 22, 1085 Budapest, Hungary; 3 Department of Family Care Methodology, Faculty of Health Science, Semmelweis University, Üllői Street 26, 1085 Budapest, Hungary

**Keywords:** IBD, diet, dietary habits, malnutrition

## Abstract

**Background**: Patients with inflammatory bowel disease (IBD) often follow restrictive diets. Data on dietary habits and beliefs in pediatric IBD are scarce. Our aim was to assess dietary habits, beliefs, and knowledge regarding nutrition and IBD among children with IBD. **Method**: In this single-center, cross-sectional study, pediatric patients aged 12–18 years with IBD completed a non-scoring, 25-item questionnaire assessing general dietary habits, beliefs about diet’s role in IBD pathogenesis and treatment, food avoidance, and food-related experiences since diagnosis. **Results**: We included 72 IBD patients (mean age [SD]: 15.2 [2.3] years; 41 (57%) were male, 32 (45%) had Crohn’s disease (CD)). Almost half of the participants did not believe that eating habits contributed to the pathogenesis of IBD. One-third of patients considered diet to be more important than medication. Approximately two-thirds changed their eating habits after diagnosis, regardless of disease type (χ^2^: 0.70, *p* = 0.40), or induction therapy (nutritional vs. non-nutritional, χ^2^: 0.02, *p* = 0.88). At least one food was avoided by 65% of the participants. **Conclusions**: Most children have changed their dietary habits and avoided one or more food groups. The type of induction therapy did not relate to the knowledge and dietary beliefs after induction. Our findings suggest that repeated education and dietary counseling should be an integral part of the management of pediatric IBD.

## 1. Introduction

Inflammatory bowel disease (IBD) is a chronic gastrointestinal condition with a variety of intestinal and extraintestinal manifestations. With a worldwide increasing incidence, IBD occurs in genetically predisposed individuals. However, the precise etiology is still uncertain [1]. According to the most recent literature, the unbalanced, interacting environment–genes–immune system triangle is the foundation of IBD [2,3]. Among the different environmental factors, diet plays a significant role in the pathogenesis of IBD [2,4,5], as the Western diet has already been linked to an increased incidence of IBD [6].

In addition to its etiological role, diet is increasingly employed as a therapeutic tool in IBD management. Exclusive enteral nutrition (EEN) is a key component of first-line treatment in pediatric CD [7]. More recently, the Crohn’s disease exclusion diet combined with partial enteral nutrition (CDED + PEN) has emerged as an alternative option, improving palatability and adherence compared with EEN alone [8]. Patients and their families are often exposed to a variety of unproven dietary beliefs, despite the established relevance of these organized nutritional therapies in disease management.

Patients with IBD often have strong personal beliefs that certain foods cause or exacerbate their symptoms, which has a substantial impact on their quality of life (QoL) and food-related QoL. As a result of food-related symptoms, many patients with IBD begin dietary changes and restrict certain types of food to manage their symptoms. Additionally, they steer clear of eating-related social encounters, which leads to social isolation, tension, and anxiety [9]. Frequent relapses, worse QoL, and dietary deficits are linked to impaired food-related QoL [10].

The uncontrolled restrictions and food avoidance have a marked impact on growth and development in the pediatric population. IBD affects not just the eating habits of young patients but also those of their parents and other relatives [11]. As a result, doctors and the caregiver team must control the symptoms associated with the illness, prevent complications (such as growth and malnutrition), and work to restore normal psychosocial functioning [9,12,13].

Gaining a better understanding of how children with IBD perceive and comprehend diet in relation to their illness is crucial for enhancing nutritional counseling and preventing needless, unbalanced diets and associated malnutrition, given the significant impact of dietary beliefs and behaviors on pediatric patients and their families [11,13]. However, there is still a dearth of pediatric literature on this subject. While direct evaluation of adolescents’ own knowledge and beliefs regarding the role of nutrition in IBD is still rare, prior research on dietary beliefs and food avoidance in pediatric IBD has mostly relied on parental reports or has concentrated on restrictive eating behaviors and food avoidance patterns [11,14,15]. In the current cross-sectional study, we sought to close this gap by evaluating dietary beliefs and knowledge about nutrition and IBD among adolescents with IBD at a tertiary pediatric clinic.

## 2. Materials and Methods

Ethical approval was obtained from the Scientific and Research Ethics Committee of the Medical Research Council (BM/12402-1/2024). Written informed consent was given by all patients and legal guardians for participation and anonymous data collection. Our survey conforms the ethical guidelines of the Declaration of Helsinki, updated in 2013. This cross-sectional study was reported in accordance with the Strengthening the Reporting of Observational Studies in Epidemiology (STROBE) statement [16].

### 2.1. Patient Sample, Data Collection and the Questionnaire

Our single-center, cross-sectional study using a brief paper-based questionnaire was performed among pediatric IBD patients from Heim Pal National Pediatric Institute, Budapest, Hungary, between December 2023 and April 2025. Inclusion criteria were as follows: age 12–18 years; an established diagnosis of IBD; and signed informed consent. Exclusion criteria were intellectual disability, ongoing EEN, and newly diagnosed IBD (within 2 months). No formal sample size calculation was performed a priori; all eligible outpatients attending regular follow-up visits during the study period were invited to participate. A total of 77 eligible outpatients who fit the inclusion criteria were contacted during the study period. Of these, five declined to participate or did not return the questionnaire. None were excluded for incomplete questionnaires; 50% of the adolescents missed at least one question, but only one patient missed 60% of the questions. In total, 72 patients in all were enrolled and included in the final analysis.

The updated Porto criteria were used to diagnose IBD [17]. Demographic and disease characteristics (age, sex, diagnosis, disease phenotype, medical therapy, diet, history of EEN and/or CDED, activity index, and body mass index (BMI) z-score) at the time of inclusion were collected from the hospital’s medical software. Disease activity was assessed using the Pediatric Crohn’s Disease Activity Index (PCDAI) and the Pediatric Ulcerative Colitis Activity Index (PUCAI) [18,19]. A suitable scale for evaluating nutritional status in the pediatric population was the BMI z-score, which was normalized for age and sex.

For our study, a short, non-validated questionnaire with two sections was applied, focusing on patients’ dietary beliefs, habits, and experiences. The disease-specific, non-scoring 25-item survey assesses dietary habits and practices and changes after the diagnosis (Supplementary Table S1). It was drafted after the literature review on this topic and modified from a previous study [20]. About 20 outpatients with IBD completed the translated and modified questionnaire for pilot testing, and the questionnaire was adjusted accordingly. The first section contained 18 single-choice questions about eating patterns, dietary habits, and knowledge. The second group of questions evaluated food intolerance/allergy and symptoms. Parents/caregivers were present in the clinic setting but were asked not to assist, and participants completed the questionnaire independently.

### 2.2. Statistical Analyses

Descriptive statistics were used to describe demographic data (sex, age, disease type, Paris classification [21], disease activity indexes, BMI z-score, and therapy). Mean and standard deviation (SD) were calculated for continuous variables, and categorical variables were presented as counts and percentages.

For questionnaire items with missing responses, participants were only excluded from the analysis of the respective item; as a result, the denominator varied across questionnaire items according to the number of available responses. Approximately half of the 72 participants omitted at least one item; item-level response rates ranged from 93% (question B.13) to 100%.

The relationship between categorical baseline characteristics and the answers was evaluated using the Chi-square test, or Fisher’s exact test, when more than 20% of cells had an expected count of less than 5, or when any cell had an expected count of less than 1; this criterion was applied both to 2 × 2 tables and to larger contingency tables. Welch’s two-sample *t*-test was used to compare continuous variables between groups. For 2 × 2 comparisons, odds ratios (OR) with 95% confidence intervals (CI) are additionally reported; for larger contingency tables, Cramér’s V is reported as a measure of effect size.

Participants were categorized into subgroups based on disease type (CD vs. UC/IBD-unclassified) and based on induction therapy in the CD subgroup (induction with or without nutritional therapy). Our cohort included CD patients diagnosed before CDED was widespread in practice; thus, we further subgrouped patients who completed at least 6-week EEN and switched to CDED, and those who completed EEN but never followed CDED (EEN vs. EEN-CDED).

Given the exploratory nature of this cross-sectional study and the large number of comparisons performed, results should be interpreted as hypothesis-generating rather than confirmatory.

The results were considered significant if *p* < 0.05. For statistical analyses, Microsoft Excel and R (version 3.5.0) were used.

## 3. Results

### 3.1. Characteristics of Study Participants

**Figure 1:**
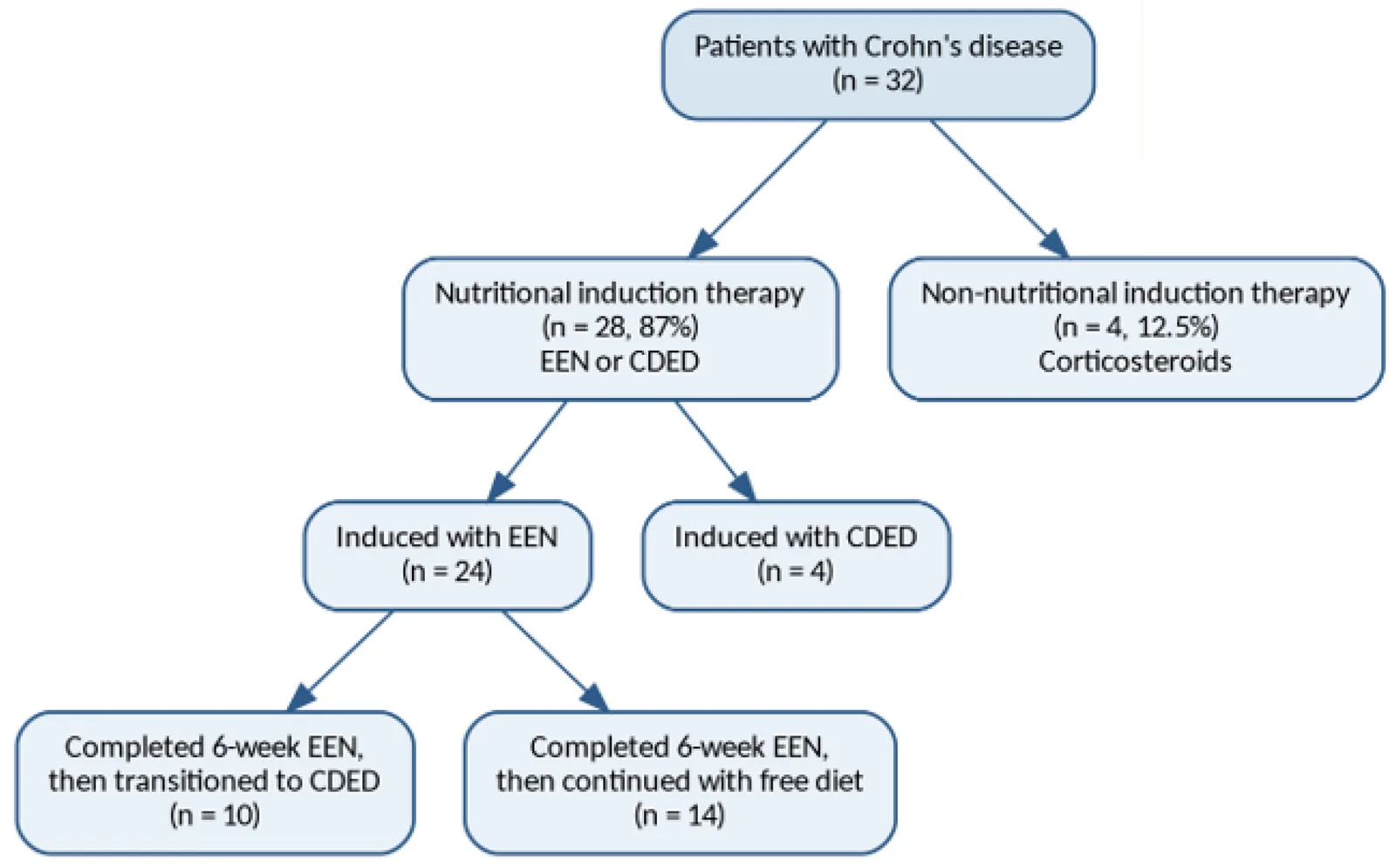
Treatment flow diagram of patients with Crohn’s disease by induction therapy.

Of the 72 participants, 41 were male (57%), 32 had CD (45%), and the mean age (±SD) was 15.2 ± 2.3 years. Most patients were in remission at the time of the study (UC: 75%, CD: 86%). Of the 72 patients, 32 (44%) received biological therapy, and 34 (47%) were on azathioprine, while 11 (15%) received corticosteroid therapy. (Table 1) Of CD patients, 28 (87%) were induced with dietary therapy (24 patients with EEN and four patients with CDED), and four (12.5%) were on steroid therapy. After a successful 6-week EEN therapy, 10 patients continued with CDED; the other 14 patients started a free diet (Figure 1).

Only one patient followed a special diet due to religion; three had celiac disease. No other condition that requires a special diet (e.g., documented food allergy or diabetes mellitus) occurred.

### 3.2. Disease and Dietary Beliefs

The first question assessed patients’ knowledge regarding the relationship between diet and IBD. Only a small proportion of patients knew that diet has an important role in the pathogenesis of IBD (*n* = 7). Almost half of the responders (46%, *n* = 34) did not believe that eating habits play an important role in the pathogenesis of IBD. A considerable proportion (42%) of the patients did not have a firm opinion on this question. More patients with CD thought that there is an association between the pathogenesis and diet than patients with UC (UC/IBD-U vs. CD, 39% vs. 61%), though no statistically significant association was observed between this response and disease type (χ^2^: 0.72, *p* = 0.69, Cramér’s V = 0.10) or sex (χ^2^: 1.41, *p* = 0.49, Cramér’s V = 0.14).

While nearly half of the participants did not attribute any importance to diet in the pathogenesis of IBD, 67% of children (*n* = 48) reported changing their eating habits to avoid symptoms after diagnosis. There was no significant association between this answer and disease type (OR 1.53, 95% CI 0.56–4.18; χ^2^: 0.70, *p* = 0.40) and sex (OR 0.58, 95% CI 0.21–1.62; χ^2^: 1.09, *p* = 0.30). Regardless of the type of disease, 45% of patients (*n* = 32) agreed that their symptoms were relieved with new eating habits (OR: 2.02, 95% CI 0.77–5.32; χ^2^: 2.05, *p* = 0.15). It is of note that those children who changed their diet significantly more often perceived an association between their symptoms and diet (OR: 0.19, 95% CI 0.03–1.22; χ^2^: 6.09, *p* = 0.0135). Furthermore, one third of patients thought diet was more important than medication, while 57% of patients (*n* = 41) believed them equivalent. No statistically significant association was found between the rate of these answers and disease type (OR: 1.38, 95% CI 0.42–4.53; χ^2^: 0.39, *p* = 0.53) or sex (OR: 2.67, 95% CI 0.90–7.90; χ^2^: 3.22, *p* = 0.073) in this sample.

### 3.3. Dietary Habits

According to the survey, nine of the 72 participants (13%) kept CDED, and 27 out of the 72 participants (38%) followed a special diet. A gluten-free diet was followed by 11% of the study participants (*n* = 8); however, only 4% of the patients (*n* = 3) had celiac disease, and only one child claimed that gluten causes symptoms. Additionally, 27% (*n* = 20) of the study group were on a lactose-free diet; among them, 55% experienced symptoms. The others followed this diet due to the physician’s (25%) or relatives’ advice (20%). The frequency of a lactose-free diet did not differ significantly between patients with CD and UC. Furthermore, 7% (*n* = 5) of the patients excluded dairy products, and 3% (*n* = 3) left ultra-processed or spicy food and food with yeast from their diet. From the whole population, 32% used dietary supplements; most of them were multivitamins or vitamin complexes.

### 3.4. Excluded Food Groups, Reason for Exclusion, Resources

**Figure 2:**
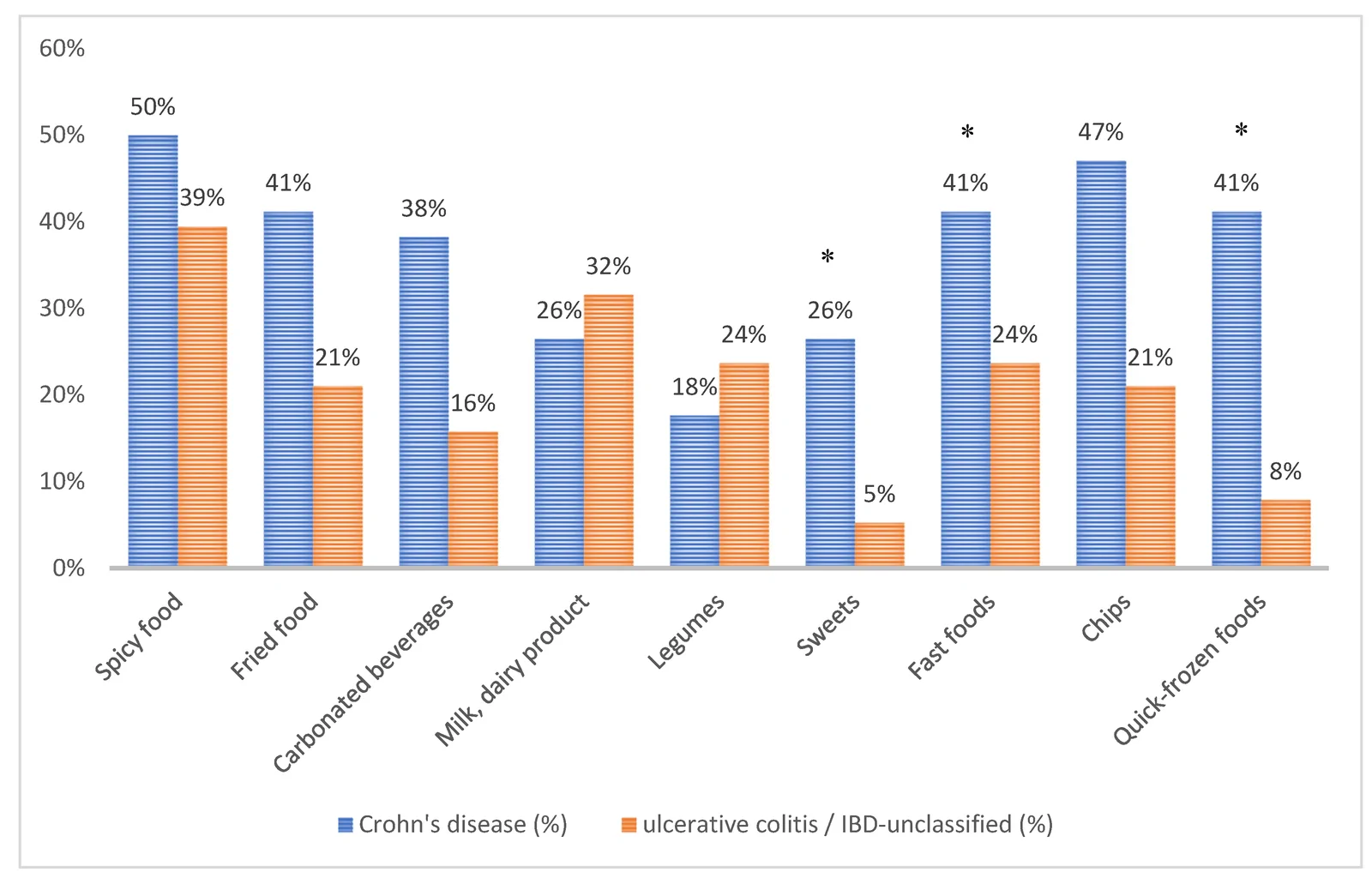
Most commonly avoided foods in patient groups. * significant results (*p* < 0.05).

At least one food was avoided by 65% of the responders (*n* = 47). However, only one third (29%) of the participants claimed that there is at least one food/drink that is worsening their symptoms. Neither food avoidance (OR = 1.49, 95% CI 0.54–4.11, χ^2^: 0.59, *p* = 0.44; 0.42, 95% CI 0.15–1.21, χ^2^: 2.66, *p* = 0.10), nor the food avoidance only at flare up was associated with disease type or sex (OR = 1.05, 95% CI 0.19–5.83, OR = 1.05, 95% CI 0.19–5.83, χ^2^: 0.00, *p* = 0.96; χ^2^: 0.00, *p* = 0.95). The list of the most common excluded foods in the CD, UC/IBD-U, and EEN, EEN-CDED subgroups is presented in Table 2. Patients with CD were significantly more likely to avoid sweets (OR 6.48, 95% CI 1.29–32.6; χ^2^ = 0.12, *p* = 0.013), chips (OR 3.33, 95% CI 1.19–9.35; χ^2^ = 4.35, *p* = 0.037), and quick-frozen foods (OR 8.17, 95% CI 2.09–31.9; χ^2^ = 9.25, *p* = 0.002) than patients with UC; no significant difference was observed for fast food avoidance (OR 2.26, 95% CI 0.82–6.21; χ^2^ = 1.79, *p* = 0.11) (Figure 2). Detailed data on avoided food groups and reasons for avoidance are shown in Supplementary Table S2.

Food avoidance can be associated with malnutrition; however, there was no statistically significant difference in BMI z-score between children who avoided at least one food and patients without any dietary restriction (mean difference 0.13, 95% CI −0.60 to 0.86; *p* = 0.73).

The main source of dietary advice was the family (51%), followed by the gastroenterologists (38%), while 29% of the participants obtained dietary information from the internet. Only 22% of patients had at least one consultation with dietitians, and of these, 81% marked it as helpful; however, these useful consultations were not repeated in 62% of the cases due to different reasons, and only 6% of the patients talked regularly with a dietitian.

### 3.5. Dietary Habits and Beliefs in the Subgroups of CD Patients Based on Their Initial Nutritional Therapy

We compared the dietary knowledge and beliefs in different subgroups based on induction treatment in patients with CD.

First, children with nutrition induction (EEN and/or CDED) and children with non-nutrition induction (28 vs. 4, respectively) were analyzed. Fewer children in the nutrition group claimed that they did not know whether diet had a role in the pathogenesis of IBD than children with non-nutrition induction. Dietary changes after the diagnosis showed no statistically significant association with the type of induction therapy (OR 0.83, 95% CI 0.08–9.25, *p* = 1.00) (Table 3).

Next, patients who completed EEN and did not meet with CDED were compared to children who were exposed to CDED after EEN (14 vs. 10, respectively). No statistically significant differences were observed between the subgroups, as seen in the detailed results in Table 4.

## 4. Discussion

In this single-center, cross-sectional study, we evaluated dietary habits and beliefs of our pediatric IBD patients. The majority of children were either unsure or did not think diet plays a role in the pathophysiology of IBD. However, 87.5% of them thought that diet is at least as important as their medication, and 65% of the participants excluded one or more foods or food groups from their diet. Patients with CD tended to report avoiding fast food and quick-frozen foods more frequently than children with UC/IBD-U, although this difference was not statistically significant. Despite the frequent food restriction, in this small cohort we did not find an association between food restriction and BMI z-score. Notably, only a small percentage of the patients had access to routine dietitian consultations.

This discrepancy between the high frequency of dietary modifications and food avoidance and the lack of understanding of the function of diet is probably due to factors other than objective nutritional decisions that influence dietary habits.

Chronic, unpredictable symptoms may drive patients toward restrictive eating as a coping strategy or an attempt to regain control over their disease; consistent with this, 60% of participants reported feeling better when paying attention to their diet. Notably, family was the most often mentioned source of dietary advice in our cohort (51%), surpassing gastroenterologists (38%) and the internet (29%). This suggests that family and social environments, rather than expert advice, influence dietary decisions in children with IBD. This tendency was also reflected in specific dietary choices: among the 27% (*n* = 20) of individuals who maintained a lactose-free diet, only 55% reported that this was based on symptom experience, while the remaining participants followed the diet on the advice of a physician (25%) or relatives (20%). These findings suggest that non-professional and family sources of dietary decisions are usually not evidence-based. Family dynamics likely play a particularly important role in this process, as parents of children with IBD have themselves been shown to hold strong dietary beliefs that influence household food practices [15]. When considered collectively, these findings suggest that dietary counseling in pediatric IBD should address patients’ knowledge gaps as well as the psychological and social factors that influence their eating behavior, such as family influence, non-professional information sources, and diet-related feelings of symptom control.

Regardless the scientific knowledge, our data showed that more than two thirds of the participants did not consider dietary habits to play an important role in the etiology of IBD [2,4,5] in line with previous findings [14,22] and with a broad range of data in the adult literature, where less than fifty percent of patients linked diet as a triggering factor of IBD [6,20,23,24,25].

This lack of firm belief in a causal role for diet did not differ by disease type, contrary to our initial hypothesis that patients with CD receiving nutritional induction therapy (EEN and/or CDED) would show greater awareness of the diet-disease relationship, this lack of firm belief in a causal role for diet did not differ by disease type. This finding, along with our subgroup analysis, suggests that nutritional induction therapy alone does not translate into lasting disease-related dietary knowledge, underscoring the need for structured, repeated education independent of treatment modality.

Similar to previous studies, almost two-thirds of the participants changed their diet after diagnosis, regardless of disease type or gender [6,24]. Interestingly, Bramuzzo et al. found a much lower rate (36%) among pediatric patients [14]. This difference may reflect differences in study population, questionnaire design, or cultural and healthcare context between cohorts. However, only half of the cases claimed that the new eating habits relieved their symptoms.

Our study revealed another common problem of patients with IBD. Only one-fifth of patients had a consultation with dietitians, close to the rate (12%) reported by Bramuzzo et al. [14]. Despite the usefulness of the consultation, they were not repeated in more than half of the cases, likely reflecting the lack of IBD-specialized dietitians in Hungarian pediatric care; however, for comprehensive patient care, dietitians should be members of everyday patient care [26,27]. The main sources of dietary advice in our study were the family, friends, and the internet, as this is a common finding of several studies [14,24,28,29].

As documented in the literature [20,24], over two-thirds of the participants valued food and nutrition equally or more than any medicine, despite the low percentage of patients believing diet plays a key role in IBD pathogenesis. Our findings are consistent with the literature [23,30,31,32], confirming that patients believe nutrition has a significant role in managing their symptoms.

Nearly two-thirds of the participants eliminated one or more foods from their diet, and every third adolescent followed a restrictive diet regardless of illness activity, even though only one-third of patients connected specific foods with increasing symptoms. We were unable to formally analyze the relationship between disease activity and food avoidance in our cohort due to the small number of patients with active disease at the time of survey completion; however, other studies have reported that disease activity influences dietary restriction, with patients with active disease practicing stricter food avoidance than those in remission [33,34]. In line with our results, the most adopted exclusion diets are gluten- or lactose-free ones in previous studies [20,24,25]. Patients with IBD are frequently advised to avoid dairy products or lactose without any diagnostic test to relieve symptoms. The percentage of patients adhering to a lactose-free diet was similar to that of previous research [20,25]. A gluten-free diet is also commonly recommended for patients with IBD. Data on the frequency of gluten-free diets is conflicting; in some studies, it is low [20], while in others it is similar to our finding [25]. Previously, a low-fiber diet was general advice in IBD, though nowadays it is encouraged only in special situations (e.g., withstricturing CD). Contrary to earlier research that found poor fiber intake [20,23], none of our patients reported avoiding fruit or vegetables (apart from legumes), and only a small percentage of patients avoided whole-grain baked items. Some dietary approaches, such as CDED, may have benefits, but unknown and uncontrolled restrictive diets could be the major cause of mineral and nutritional deficiencies [35]. However, in our cohort, none of the children were undernourished based on their BMI z-scores [20,36]. Our results highlight that patients need professional discussion on their diet to ensure a balanced diet.

To evaluate the impact of nutrition induction therapy and the related education, we compared dietary beliefs and knowledge in different subgroups. Although the low number of cases means a severe limitation to drawing conclusions, it seems that following CDED was not related to more knowledge on the relationship between diet and IBD. Our data suggest that patients receiving nutrition therapy still need repeated education on the role of diet in IBD.

This study has several strengths and weaknesses. Available data on this topic with pediatric IBD patients are limited; our study involved one of the largest pediatric IBD populations [9,14,37]. Participants were recruited consecutively from a tertiary referral center; demographic and clinical data were collected from standardized medical records. The gender and disease type ratio was nearly half; our patient group was homogenous (ethnic origin, food culture). In addition, questionnaire administration was standardized, reducing variability in data collection. Several limitations should also be considered when interpreting the findings. As this study involved numerous exploratory comparisons without correction for multiple testing, our findings should be interpreted as hypothesis-generating, and confirmation through larger, adequately powered studies is warranted. The single-center tertiary care design and the relatively small sample size may increase the risk of selection and referral bias. Subgroup analyses (e.g., based on disease activity) were limited by small sample sizes. Although all participants returned the questionnaire and missing data was infrequent, approximately half of the participants omitted at least one item. Item-specific missing responses may have introduced non-response bias if unanswered questions systematically differed from completed ones. Cross-cultural validation, introducing the possibility of measurement bias, was not performed. Furthermore, information bias may also occur due to inaccurate recall or social desirability, particularly regarding dietary habits and food avoidance. Despite the significant parental influence, their perspectives were not assessed; however, the dietary practices in these age groups may partly reflect parental beliefs rather than the adolescents’ own views.

## 5. Conclusions

In conclusion, most children with IBD changed their diet after the diagnosis, and about one-third of them adhered to a special diet that may not have been medically indicated. Furthermore, patients with CD predominantly avoided ultra-processed food, whereas patients with UC primarily avoided foods perceived to worsen symptoms. Teenagers’ ignorance of diseases emphasizes how crucial it is to comprehend patients’ beliefs to enhance the standard of care. This is the first study evaluating the association between nutritional induction therapy and subsequent dietary beliefs in pediatric IBD, and we did not identify such an association in our cohort. As part of standard IBD care, our findings support the use of frequent, age-appropriate nutritional education and repeated dietary counseling. To dispel myths, avoid needless food restrictions, and advance balanced nutrition, a multidisciplinary approach combining pediatric gastroenterologists and dietitians should be promoted.

Future studies would benefit from a multicenter design with larger pediatric IBD cohorts, employing validated questionnaires and incorporating parental input to allow direct comparison between adolescents’ and parents’ perspectives. While a detailed assessment of dietary intake and nutritional outcomes was beyond the scope of the present study, future work combining these approaches could help link patients’ beliefs and behaviors to objective nutritional status.

## Figures and Tables

**Table 1:** The main characteristics of patients involved.

Characteristics	Analyzed Population (*n* = 72)	
Male (%)	41 (57)	
Age (mean ± SD; years)	15.2 ± 2.3	
CD/UC, IBD-U (*n*)	32/40	
Paris classification (*n*, %)	CD	UC
	L1: 7 (19) L1L4: 6 (16) L2: 4 (11) L2L4: 5 (13) L3: 6 (16) L3L4: 8 (22) isolated L4: 1 (3)	E1: 6 (17) E2: 6 (17) E3: 8 (23) E4: 15 (43)
PCDAI (mean ± SD)	4.0 ± 6.98	
PUCAI (mean ± SD)	4.3 ± 7.1	
BMI z-score (mean ± SD)	−0.3 ± 2.6	
CD induction therapy (%):		
EEN	24 (75)	
CDED	4 (12.5)	
Current therapy		
azathioprine	34 (47)	
corticosteroid	11 (15)	
5-ASA	35 (49)	
biological therapy	32 (44)	

CD: Crohn’s disease; UC: ulcerative colitis; IBD-U: inflammatory bowel disease, unclassified; PCDAI: Pediatric Crohn’s Disease Activity Index; PUCAI: Pediatric Ulcerative Colitis Activity Index; EEN: exclusive enteral nutrition; CDED: Crohn’s disease exclusion diet; 5-ASA: 5-aminosalicylate. Paris classification: L1: distal 1/3 ileal ± limited cecal disease; L2: colonic; L3: ileocolonic; L4a: upper gastrointestinal disease-proximal to the ligament of Treitz; L4b: upper gastrointestinal disease-distal to the ligament of Treitz and proximal to distal 1/3 ileum. E1: ulcerative proctitis; E2: left-sided UC (distal to splenic flexure); E3: extensive (hepatic flexure distally); E4: pancolitis (proximal to hepatic flexure).

**Table 2:** The five most commonly avoided food groups in Crohn’s disease and ulcerative colitis.

UC/IBD-U (*n* = 40)	Crohn’s Disease (*n* = 32)	EEN (*n* = 14)	EEN + CDED (*n* = 10)
Hot, spicy (42%)	Spicy (50%)	Spicy (43%)	Spicy (80%)
Spicy (39%)	Chips (47%)	Fried food (43%)	Chips (80%)
Milk, dairy products (32%)	Quick-frozen food (41%)	Processed meats (36%)	Quick-frozen food (70%)
Fast food (24%)	Fried food (41%)	Fast food (36%)	Fried food (70%)
Legumes (24%)	Fast food (41%)	Quick-frozen food (36%)	Sweets (70%)

UC: ulcerative colitis; IBD-U: inflammatory bowel disease unclassified; EEN: exclusive enteral nutrition; CDED: Crohn’s disease exclusion diet.

**Table 3:** Comparison of patients with nutrition induction vs. non-nutrition induction.

	Nutrition Induction (*n* = 28)	Non-Nutrition Induction (*n* = 4)	*p*-Value (OR/CI or Cramer’s V)
Male (%)	17 (60.1)	2 (50.0)	
Age (mean ± SD; years)	15.1 ± 2.2	17.2 ± 0.9	
PCDAI (mean ± SD)	4.0 ± 7.0	1.3 ± 2.2	
BMI z-score (mean ± SD)	−0.9 ± 2.7	1.9 ± 3.4	
Diet has an important role in the pathogenesis of IBD			*p* = 1.0 ^†^ (Cramér’s V = 0.13)
Yes, *n* (%)	3 (11) *	0 (0.0)	
No, *n* (%)	11 (39)	2 (50.0)	
Do not know (%)	13 (46)	2 (50.0)	
Diet changes after the diagnosis			*p* = 1.0 ^†^ (OR 0.83, 95% CI 0.08–9.25)
Yes, *n* (%)	20 (71.4)	3 (75.0)	
No, *n* (%)	8 (28.57)	1 (25.0)	
Diet is more important than medicine			-
More important, *n* (%)	11 (39.3)	0 (0.0)	
Equivalent, *n* (%)	16 (57.1)	3 (75.0) *	
Less important, *n* (%)	0 (0.0)	0 (0.0)	
Not important, *n* (%)	1 (3.6)	0 (0.0)	

PCDAI: Crohn’s disease activity index; BMI: body mass index; IBD: inflammatory bowel disease. * incomplete participation, missing answer. ^†^ Fisher’s exact test.

**Table 4:** Comparison of the Crohn’s disease patients: completed exclusive enteral nutrition followed by free diet vs. completed exclusive enteral nutrition followed by Crohn’s disease exclusive diet.

Baseline Data and Answers	EEN (*n* = 14)	EEN + CDED (*n* = 10)	Significance
Male (%)	9 (64)	5 (50)	
Age (mean ± SD; years)	15.8 ± 1.8	13.3 ± 2.1	
PCDAI (mean ± SD)	3.2 ± 4.0	5.0 ± 7.1	
BMI z-score (mean ± SD)	−0.9 ± 3.3	−0.2 ± 0.9	
Diet has an important role in the pathogenesis of IBD:			*p* = 1.0
Yes, *n* (%)	1 (7)	1 (10)	
No, *n* (%)	6 (43)	4 (40)	
Do not know (%)	6 (43)	5 (50)	
Diet changes after the diagnosis			*p* = 0.21
Yes, *n* (%)	9 (64)	9 (90)	
No, *n* (%)	5 (36)	1 (10)	
Diet is more important than medicine			*p* = 0.63
More important, *n* (%)	6 (43)	4 (67) *	
Equivalent, *n* (%)	8 (57)	2 (33)	
Less important, *n* (%)	0 (0)	0 (0)	
Not important, *n* (%)	0 (0)	0 (0)	

EEN: Exclusive enteral nutrition; CDED: Crohn’s disease exclusive diet; PCDAI: Crohn’s disease activity index; BMI: body mass index; IBD: inflammatory bowel disease. * incomplete participation, missing answer.

## Data Availability

The datasets used and analyzed during the current study are available from the corresponding author upon reasonable request.

## Supplementary Materials

The following supporting information can be downloaded at https://www.mdpi.com/article/10.3390/nu18162675/s1. Table S1: Dietary habits and beliefs questionnaire; Table S2: The reasons of food avoidance.

## Abbreviations

IBD	Inflammatory bowel disease
QoL	Quality of life
CD	Crohn’s disease
UC	Ulcerative colitis
EEN	Exclusive enteral nutrition
CDED	Crohn’s disease exclusion diet
