# Supplementary material for: Dietary Beliefs and Habits in Pediatric IBD: Insights from a Single-Center Survey
Source: Nutrients. 2026 Aug 16;18(16):2675. doi: 10.3390/nu18162675 (PMC13516628; doi:10.3390/nu18162675)
Supplement: Supplementary file 1 [file nutrients-18-02675-s001.zip › Supplementary Table S1_revision 08.pdf]

Supplementary Table S1 Dietary habits and beliefs questionnaire

Date_____________________________

A) Dietary habits

A.1) Do you think, your eating habits could cause your illness?

□ yes □ no □ I do not know

A.2) Did you consultate with a dietitian after the diagnosis?

□ yes □ no

- If yes, was it useful? □ yes □ no
- If yes, do you have regular consultation with him/her? □ yes □ no

A.3) Who do you regularly ask for dietary advice?

□ internet/book □ doctor □ dietitians □ family □ friend □ other: _______________

A.4) Did you change your eating habits after the diagnosis?

□ yes, what: _______________ □ no

A.5) Did you notice any connection between your changed eating habits and your symptoms?

□ yes □ no

A.6) Did you change your eating habits after biological therapy induction?

□ yes □ no

A.7) Are you on any special diet? (e.g. gluten-free, diary product free, CDED)?

□ yes □ no

- If yes, what is it? _______________

A.8) Is your religion influence your eating habits?

□ yes □ no

- If so, what principles do you follow? ____

A.9) How many times do you eat a day?

□ 2 □ 3 □ 4 □ 5 □ 6 □ 7 □ haphazardly □ several times, a little □ variable

A.10) Do you eat just as often during a relapse as you do in remission.?

□ yes □ no □ I have not any relapse yet

A.11) Do you use any dietary supplements?

□ yes □ no

- If so, what and which brand? ______________

A.12) Do you consume formula daily, alongside your meals?

□ yes □ no

- If so, how much? (e.g: 1 bottle, 200 ml) _____________

A.13) Are there any food or drink which worsening your symptoms?

□ yes □ no

A.14) Have you avoided any food since the diagnosis?

□ yes □ no

A.15) Do you avoid these foods always or just during a relapse?

□ yes, always □ no, only at relapse

A.16) What is your opinion? Compared to medication, proper and healthy nutrition…

□ more important □ equal □ less important □ not important

A.17) Do the medications influence your appetite?

□ yes, I am hungrier □ yes, I am less hungry □ no, it is not influence anything

A.18) Which of the listed foods do you like, and which do you avoid?

	I do not like	Avoiding due to symptoms worsening	Consuming despite symptoms	Avoiding on my doctor's recommendation	Consuming without problem
hot, spicy foods	□	□	□	□	□
spicy foods	□	□	□	□	□
fried foods	□	□	□	□	□
carbonated beverages	□	□	□	□	□
milk and dairy products	□	□	□	□	□
energy drinks	□	□	□	□	□
alcohol	□	□	□	□	□
pork	□	□	□	□	□
processed foods	□	□	□	□	□

vegetables	□	□	□	□	□
fruits	□	□	□	□	□
legume	□	□	□	□	□
whole grain bakery products	□	□	□	□	□
bread	□	□	□	□	□
egg	□	□	□	□	□
rice	□	□	□	□	□
chicken	□	□	□	□	□
noodle	□	□	□	□	□
fish	□	□	□	□	□
coffee	□	□	□	□	□
sweets	□	□	□	□	□
fast food (pl.: pizza, hamburger)	□	□	□	□	□
chips	□	□	□	□	□
quick-frozen foods	□	□	□	□	□

B) Food intolerance, allergy

B.1) Are you on a gluten-free diet?

□ yes □ no

- If so, why?

□ consuming gluten causes problems
□ doctor suggested
□ dietitian suggested
□ friend/ family suggested
□ I have coeliac disease
□ other ________________________________

B.2) Which of the following symptoms do you experience after consuming gluten?

□ no symptoms
□ fullness feeling
□ abdominal pain
□ loss of appetite
□ diarrhoea
□ obstipation
□ burping
□ bloating
□ weakness
□ nausea/vomiting
□ other, name it ________________________

B.3) Are you on a lactose free diet?

□ yes □ no

- If so, why?

□ consuming lactose causes problems
□ doctor suggested

□ dietitian suggested
□ friend/ family suggested
□ other, name it ________________________

B.4) Which of the following symptoms do you experience after consuming lactose?

□ no symptoms
□ fullness feeling
□ abdominal pain
□ loss of appetite
□ diarrhoea
□ obstipation
□ burping
□ bloating
□ weakness
□ nausea/vomiting
□ other, name it ________________________

B.5) Do you keep any other diet?

□ yes □ no

- If so, which one? _______________ And why do you keep it?

□ because its consumption causes symptomsdoctor suggested
□ dietitian suggested
□ friend/ family suggested
□ my parents chose it
□ other, name it ________________________

B.6) Which of the following symptoms do you experience after consuming it?

□ no symptoms
□ fullness feeling
□ abdominal pain
□ loss of appetite
□ diarrhoea
□ obstipation
□ burping
□ bloating
□ weakness
□ nausea/vomiting
□ other, name it ________________________

B.7) What do you think, if you pay attention to your diet, do you feel better and less sick?

□ yes □ no
