# Supplementary material for: Dietary Beliefs and Habits in Pediatric IBD: Insights from a Single-Center Survey
Source: Nutrients. 2026 Aug 16;18(16):2675. doi: 10.3390/nu18162675 (PMC13516628; doi:10.3390/nu18162675)
Supplement: Supplementary file 1 [file nutrients-18-02675-s001.zip › Supplementary Table S2_revision 08.pdf]

Supplementary Table S2 The reasons of food avoidance

	Avoiding due to symptoms worsening		*Avoiding due to physician' advice*		*Consuming without problem*		*Consuming despite symptoms*	
	CD	UC	CD	UC	CD	UC	CD	UC
hot, spicy foods, n (%)	5 (16)	11 (29)	8 (25)	5 (13)	8 (25)	11 (29)	1 (3)	0 (0)
spicy foods, n (%)	8 (25)	6 (15)	10 (31)	9 (22)	11 (34)	21 (53)	0 (0)	1 (3)
fried foods, n (%)	6 (19)	2 (5)	9 (28)	6 (15)	15 (47)	27 (69)	1 (3)	2 (5)
carbonated beverages, n (%)	4 (13)	4 (10)	9 (28)	2 (5)	16 (50)	25 (64)	0 (0)	2 (5)
milk and dairy products, n (%)	5 (16)	9 (24)	4 (13)	3 (8)	19 (61)	22 (58)	0 (0)	1 (3)
energy drinks, n (%)	3 (10)	3 (8)	4 (13)	3 (8)	4 (13)	8 (22)	0 (0)	0 (0)
alcohol, n (%)	3 (10)	5 (14)	2 (7)	4 (11)	2 (7)	2 (6)	0 (0)	0 (0)
pork, n (%)	1 (3)	1 (3)	7 (22)	1 (3)	20 (63)	32 (82)	1 (3)	1 (3)
processed foods, n (%)	4 (13)	1 (3)	7 (23)	0 (0)	19 (61)	31 (82)	0 (0)	1 (3)
vegetables, n (%)	0 (0)	0 (0)	0 (0)	0 (0)	28 (88)	33 (85)	0 (0)	1 (3)
fruits, n (%)	0 (0)	0 (0)	0 (0)	0 (0)	28 (88)	33 (85)	1 (3)	2 (5)
legume, n (%)	1 (3)	7 (18)	5 (16)	2 (5)	17 (55)	23 (61)	0 (0)	1 (3)
whole grain bakery products, n (%)	2 (6)	3 (8)	2 (5)	0 (0)	21 (66)	24 (62)	0 (0)	1 (3)
bread, n (%)	1 (3)	2 (5)	6 (19)	0 (0)	23 (72)	33 (85)	0 (0)	1 (3)
egg, n (%)	0 (0)	1 (3)	0 (0)	0 (0)	30 (94)	33 (85)	1 (3)	1 (3)
rice, n (%)	0 (0)	0 (0)	0 (0)	0 (0)	30 (94)	36 (92)	0 (0)	1 (3)
chicken, n (%)	0 (0)	0 (0)	0 (0)	0 (0)	31 (97)	37 (93)	0 (0)	1 (3)
pasta, n (%)	0 (0)	0 (0)	2 (6)	0 (0)	29 (90)	35 (90)	0 (0)	1 (3)
fish, n (%)	0 (0)	1 (3)	0 (0)	0 (0)	21 (66)	31 (84)	1 (3)	1 (3)
coffee, n (%)	0 (0)	3 (8)	2 (6)	4 (10)	10 (31)	9 (22)	0 (0)	0 (0)
sweets, n (%)	3 (9)	1 (3)	6 (19)	1 (3)	21 (66)	31 (82)	0 (0)	2 (5)
fast foods, n (%)	8 (25)	3 (8)	7 (22)	7 (18)	14 (44)	25 (63)	1 (3)	1 (3)
chips, n (%)	6 (19)	3 (8)	10 (31)	5 (13)	15 (47)	29 (73)	0 (0)	1 (3)
quick-frozen foods, n (%)	4 (13)	2 (5)	10 (31)	1 (3)	14 (44)	26 (68)	0 (0)	1 (3)

CD: Crohn's disease; *UC:* ulcerative colitis
